# Borotropic shifting of the hydro­tris­[3-(2-furyl)pyrazol-1-yl]borate ligand in high-coordinate lan­tha­nide com­plexes

**DOI:** 10.1107/S2053229624003115

**Published:** 2024-04-16

**Authors:** Jarrod R. Thomas, Scott A. Sulway

**Affiliations:** a University of New South Wales, Sydney, NSW 2052, Australia; University of Melbourne, Australia

**Keywords:** crystal structure, lan­tha­nide, high-coordinate, borotropic shifting, icosa­hedral, dysprosium, cerium, scorpionate

## Abstract

The coordination of hydro­tris­[3-(2-furyl)pyrazol-1-yl]borate (Tp^2-Fu^, C_21_H_16_BN_6_O_3_) to lan­tha­nide(III) ions is achieved for the first time with the com­plex [Ln(Tp^2-Fu^)_2_](BPh_4_)·*x*CH_2_Cl_2_ (**1-Ln** has Ln = Ce and *x* = 2; **1-Dy** has Ln = Dy and *x* = 1). This was accom­plished *via* both hydrous (Ln = Ce) and anhydrous methods (Ln = Dy). When isolating the dysprosium analogue, the filtrate produced a second crop of crystals which were revealed to be the 1,2-borotropic-shifted product [Dy(κ^4^-Tp^2-Fu^)(κ^5^-Tp^2-Fu^*)](BPh_4_) (**2**) {Tp^2-Fu^* = hydro­bis­[3-(2-furyl)pyrazol-1-yl][5-(2-furyl)pyrazol-1-yl]borate}. We con­clude that the pres­ence of a strong Lewis acid and a sterically crowded coordination environment are contributing factors for the 1,2-borotropic shifting of scorpionate ligands in conjunction with the size of the conical angle with the scorpionate ligand.

## Abstract 2

The coordination chemistry of the hydro­tris­[3-(2-furyl)pyrazol-1-yl]borate (Tp^2-Fu^) ligand is explored with cerium(III) and dysprosium(III). The difference in Lewis acidity of the ions reveals varying stability in the *bis*-Tp^2-Fu^ lan­tha­nide com­plexes.

## Introduction

Since their genesis, scorpionate ligands have been used in coordination chemistry due to their high denticity and charged nature (Trofimenko, 1966 ▸). It was not until the 1970s that the first lan­tha­nide com­plexes featuring scorpionate ligands were synthesized, namely, LnTp_3_ [Tp = hydro­tris­(pyrazol-1-yl)bor­ate] com­plexes (Bagnall *et al.*, 1976 ▸), but since then many lan­tha­nide com­plexes have followed [there are 528 structures in the Cambridge Structural Database (CSD; Groom *et al.*, 2016 ▸) as of December 2023]. One such scorpionate that has not yet been used in lan­tha­nide coordination chemistry is hydro­tris­[3-(2-furyl)pyrazol-1-yl]borate (Tp^2-Fu^), which bears a resemblance to hydro­tris­[3-(2-pyridyl)pyrazol-1-yl)bor­ate (Tp^2-py^), where the 2-pyridyl group has been replaced by the differently coordinating 2-furyl heterocycle (Scheme 1). Tp^2-Fu^ has been used previously in transition-metal com­plexation, namely, with Cu (Halcrow *et al.*, 1997 ▸) and Zn (Maldonado Calvo *et al.*, 2006 ▸); however, the 2-furyl substituents do not partake in any coordination in these transition-metal com­plexes. The Tp^2-py^ ligand was heavily used within lan­tha­nide coordination chemistry in the late 1990s and early 2000s by McCleverty and Ward (Amoroso *et al.*, 1994 ▸; Jones *et al.*, 1997 ▸; Bell *et al.*, 2001 ▸; Beeby *et al.*, 2002 ▸), where the ligand was exploited for its hexa­dentate nature as the authors quote ‘the cavity is an appropriate size for lan­tha­nide(III) ions’ (Jones *et al.*, 1997 ▸). In McCleverty and Ward’s systems, the 2-pyridyl substituents do coordinate to lan­tha­nide ions, but systematic changes to this functional group were not explored by the authors.

Due to the difference in ring size of the 3-*R*-pyrazole (where *R* = 2-pyridyl or 2-furyl) functional groups, they present a different cavity size and, by extension, conical angles (where the measured conical angle is from the metal centre to the B atom of the scorpionate then to the coordinating N atom on the pyrazolyl ring, *i.e.* Ln⋯B⋯N_pz_), and therefore are likely 
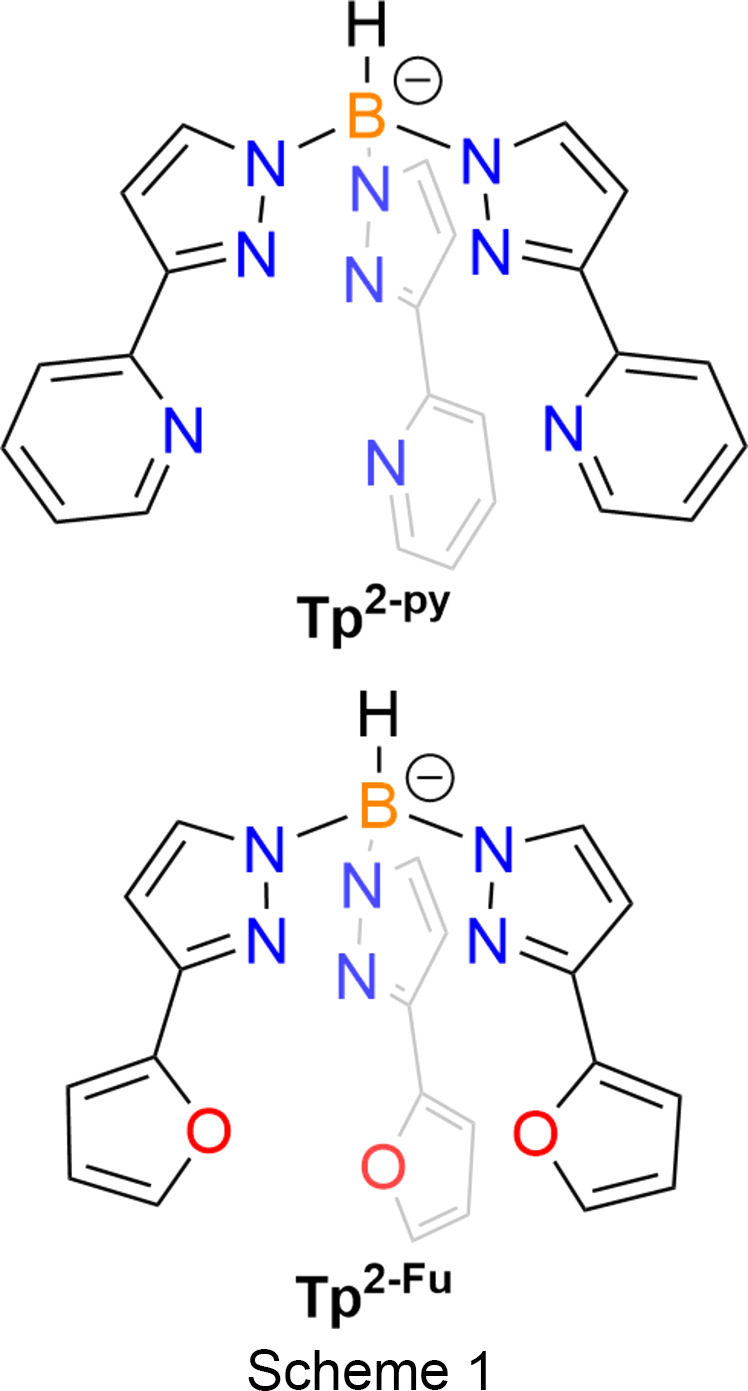
 to have different coordination chemistry. It has recently been shown by us (Thomas *et al.*, 2024 ▸) that when encapsulating lan­tha­nide ions in a *bis*-Tp^2-py^ ligand environment, the size of the lan­tha­nide ion dictates the stability of the high-coordinate com­plexes. The 12-coordinate pseudo-icosa­hedral com­plexes ([Ln(Tp^2-py^)_2_]^+^) exhibit high stability from lanthanum(III) to terbium(III) before the dysprosium(III) analogue converges on a stable 9-coordinate water-introduced com­plex [Dy(κ^4^-Tp^2-py^)_2_(H_2_O)]^+^. Thus, to explore the stability of *bis*-Tp^2-Fu^ lan­tha­nide com­plexes, the coordination chemistry of said ligand is presented herein.

## Experimental

### Synthesis and crystallization

NaTp^2-Fu^ was synthesized according to literature procedures (Halcrow *et al.*, 1997 ▸), with progression of the pyrazolyl substitution tracked by our *in-situ* methods (Thomas *et al.*, 2021 ▸). [Ce(Tp^2-Fu^)_2_](BPh_4_)·2CH_2_Cl_2_ (**1-Ce**) was prepared by an adapted literature procedure with analogous ligands (Jones *et al.*, 1997 ▸; Thomas *et al.*, 2024 ▸), *i.e.* a one-pot salt metathesis reaction in methanol from CeCl_3_·7H_2_O, NaTp^2-Fu^ and NaBPh_4_ at a 1:2:1 loading, where **1-Ce** was recrystallized from CH_2_Cl_2_/pentane. A similar literature adaptation was used to prepare [Dy(Tp^2-Fu^)_2_](BPh_4_)·CH_2_Cl_2_ (**1-Dy**), *i.e.* an anhydrous one-pot salt metathesis reaction in dry tetra­hydro­furan (THF) from anhydrous DyCl_3_, NaTp^2-Fu^ and NaBPh_4_ at a 1:2:1 loading, where **1-Dy** was recrystallized from CH_2_Cl_2_/pentane. After fil­tering the mother liquor of **1-Dy** under inert conditions, crystals of [Dy(κ^4^-Tp^2-Fu^)(κ^5^-Tp^2-Fu^*)](BPh_4_) (**2**) {Tp^2-Fu^* = hydro­bis­[3-(2-furyl)pyrazol-1-yl][5-(2-furyl)pyrazol-1-yl]bor­ate} were grown.

Full details of the syntheses are given in the supporting information.

### Single-crystal collection and refinement

Crystal data, data collection and structure refinement details are summarized in Table 1 ▸. H atoms were fixed using the riding model, apart from the borohydride H atoms (on the scorpionate ligands), which were placed based on residual electron density, with bond distances and angles allowed to refine until convergence. *OLEX2* (Dolomanov *et al.*, 2009 ▸) was employed for refining data and producing the mol­ecular graphics.

The following disorder was observed. **1-Ce** had large residual electron-density peaks which were removed with a solvent mask and assigned to an extra CH_2_Cl_2_ mol­ecule. **1-Dy** had a 3-(2-furyl)pyrazole fragment in the lattice that was removed *via* the implantation of a solvent mask using *OLEX2*, correlating to *ca* 0.75 pyrazole units. **1-Dy** also displayed a twisting disorder in the arene rings of the BPh_4_^−^ counter-ion and lattice solvent disorder for the CH_2_Cl_2_ mol­ecule. Structure **2** has a large unexplained electron-density peak close to a C atom of the BPh_4_^−^ anion. This could not be modelled as disorder and thus may indicate an unresolved problem with the reflection data.

## Results and discussion

Previous coordination of Tp^2-Fu^ or its derivatives was achieved with the copper(II) com­plex [Cu*L*(Tp^2-Fu^)]BF_4_ [*L* = 3-(2,5-di­meth­oxy­phen­yl)-1-(2-pyridyl)pyrazole] and the zinc(II) com­plexes [Zn(Tp^2-Fu,Me^)_2_] and [Zn(Tp^2-Fu,Me^)*R*] {Tp^2-Fu,Me^ = hydro­tris­[3-(2-furyl)-5-methyl­pyrazol-1-yl]borate; *R* = Cl, Br, I, NCS, CH_3_CO_2_, CF_3_CO_2_, OH, CH_3_OCO_2_, SCSOMe, OPO(ONit)_2_, ONit ­(*i.e.* 4-nitro­phen­yl), S(CH_2_)_3_COOMe or NHCOCF_3_}. The transition-metal com­plexes result in short coordination bonds with the coordinating pyrazolyl N atom [N_pz_; Cu—N_pz_ = 1.985 (4)–2.241 (4) Å and Zn—N_pz_ = 2.011 (3)–2.257 (6) Å] and, for the *bis*-capped com­plexes, the 2-furyl groups point away from the metal centres. Since the 3-*R*-substituted scorpionate ligands that employ coordinating functional groups in the past have been shown to coordinate to lan­tha­nide ions, we envisioned the same would be true for the Tp^2-Fu^ ligand.

The syntheses of **1-Ce** and **1-Dy** were achieved *via* adaptations of literature procedures (Jones *et al.*, 1997 ▸; Thomas *et al.*, 2024 ▸). Use of hydrated CeCl_3_ and NaTp^2-Fu^ stirred in methanol before the addition of a methano­lic solution of NaBPh_4_ produces a precipitate of **1-Ce**, which was further recrystallized from layering a CH_2_Cl_2_ solution of **1-Ce** with pentane. **1-Ce** is air-stable, does not decom­pose over time and has been stored open to air for over a year. Due to the size of dysprosium(III) ions, we synthesized **1-Dy** by anhydrous routes to encourage the formation of the pseudo-icosa­hedral crystal field, as the use of hydrated methods for analogous ligands results in the rearrangement of the com­plex to one with a lower coordination number (Thomas *et al.*, 2024 ▸). Thus, an­hy­drous DyCl_3_, NaTp^2-Fu^ and NaBPh_4_ were all stirred in THF before the solvent was removed *in vacuo*, and the crude material was dissolved in CH_2_Cl_2_, filtered and layered with pentane to produce single crystals of **1-Dy**.

**Figure 1:**
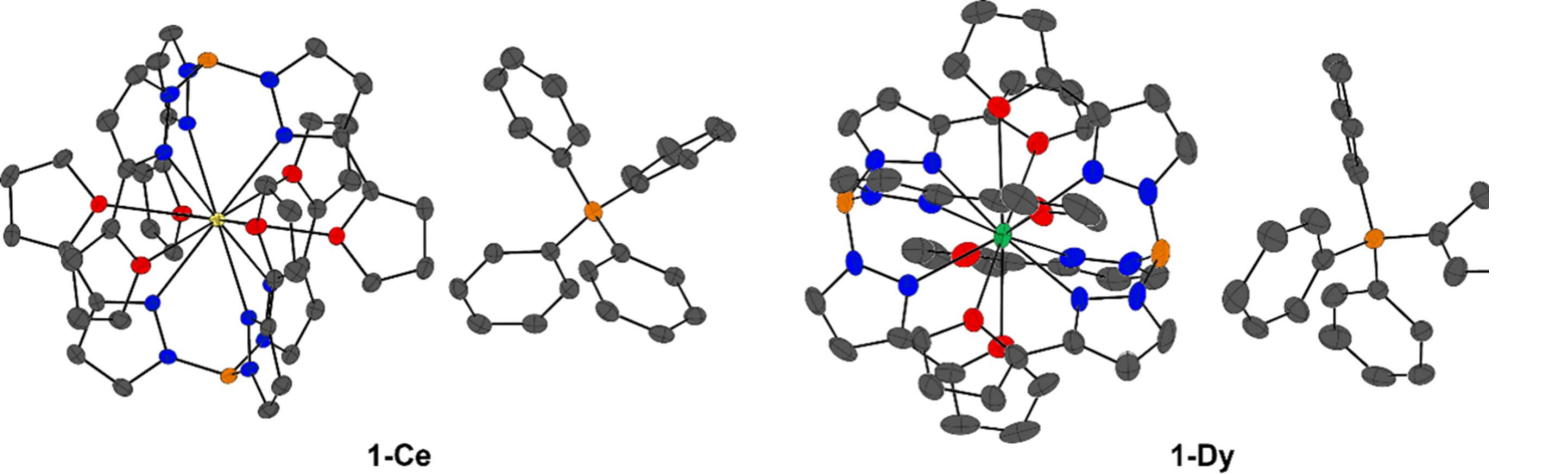
Solid-state structures of **1-Ln**, where Ln = Ce (left) and Dy (right), shown with 50% probability displacement ellipsoids. Colour codes: Dy^III^ turquoise, Ce^III^ cream, O red, N blue, C grey and B orange. H atoms have been omitted for clarity.

The solid-state structures of the cations in **1-Ce** and **1-Dy** are presented in Fig. 1 ▸, with selected data summarized in Table 2 ▸. The cations in **1-Ce** and **1-Dy** both produced pseudo-icosa­hedral (*I*_c_) crystal fields where the N_pz_ atoms are axial, akin to cyclopentadienyl (Cp) donations being axial, and with the coordinating O atoms from the furyl groups (O_Fu_) forming an equatorial ‘belt’ with respect to the N_pz_. Due to the size differences between Ce^III^ and Dy^III^, most of the coordination bonds are shorter in **1-Dy** owing to the scorpionate ligand residing closer to the lan­tha­nide centre (see Table 2 ▸ for B⋯Ln distance). Few Ln—O_Fu_ distances are larger in **1-Dy** since the furyl group extends past the plane that divides the mol­ecule in half, and there are larger torsion angles between the pyrazolyl and furyl groups within each borate substituent (torsion angle is measured from N_pz_ to O_Fu_, including the two C atoms between the N_pz_ and O_Fu_ atoms).

**Figure 2:**
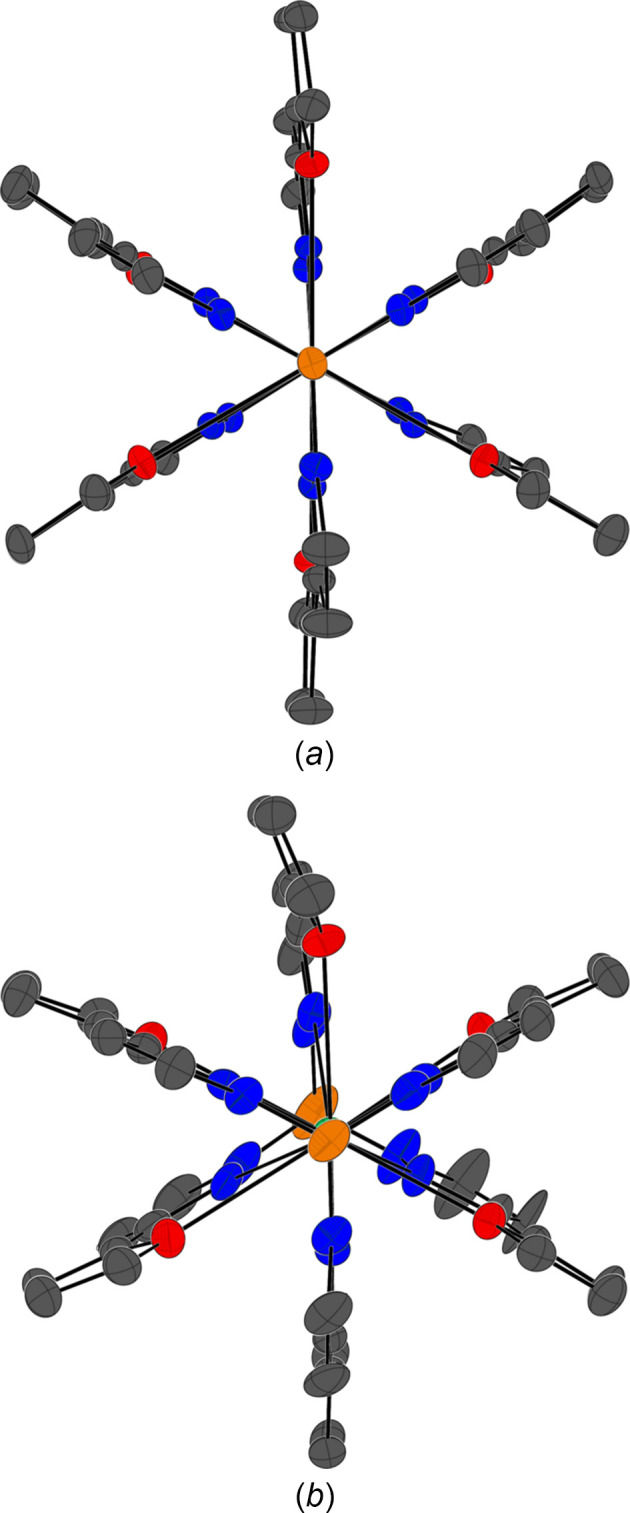
View from the pseudo-B⋯B axis in **1-Ln**, where Ln is (*a*) Ce and (*b*) Dy.

The coordination distances and measured angles in **1-Dy** have larger ranges than those observed in **1-Ce**, suggesting that the structure is sterically crowded. Steric crowding is most evident by the deviation of the B⋯Ln⋯B angle from being approximately linear in **1-Ce** to 173.1 (1)° in **1-Dy**. Viewing the cations in **1-Ln** from the pseudo-B⋯B axis (Fig. 2 ▸) brings light to said strain seen in **1-Dy**, where it can be thought that there is a loss of the psudeo-*S*_6_ axis that is more evident in **1-Ce**. These axial perspectives make it easier to visualize the larger torsion angles of the furyl groups that are present in **1-Dy**.

To investigate the strain seen in **1-Dy**, Continuous Shape Measurements (CShM) were performed employing *SHAPE2.1* (Pinsky *et al.*, 1998 ▸) to measure the distortion of the crystal field from a regular *I*_c_ (Table 2 ▸). The results of the CShM not only show that the crystal field in **1-Dy** deviates more from *I*_c_ than in **1-Ce**, but that **1-Dy** also exhibits the highest CShM value seen in pseudo-*I*_c_ *bis*-scorpionate-en­cap­sulated lan­tha­nide com­plexes (Thomas *et al.*, 2024 ▸). The high CShM value is no surprise given the strain in the cation present in Fig. 2 ▸.

Upon filtering off the mother liquor to isolate **1-Dy**, the recrystallization of 1,2-borotropic-shifted com­plex **2** occurred within the filtrate over a period of several days. Borotropic shifting in scorpionate ligands has been reported previously and occurs due to the presence of a large substituent on the third position of the pyrazolyl group, *i.e.* isopropyl (Trofimenko *et al.*, 1989 ▸; Cano *et al.*, 1990 ▸; White *et al.*, 2009 ▸), *tert*-butyl (Chisholm *et al.*, 1996 ▸), phenyl (Zhao *et al.*, 2007 ▸; Cui *et al.*, 2010 ▸) and mesityl (Rheingold *et al.*, 1993 ▸), causing steric congestion around a metal centre and hence undergoes rearrangement to reduce the steric crowding around it. One example that is noteworthy is [Nd(Tp^Ph^*)(THF)(μ-PC_6_H_3_-2,6-^*i*^Pr_2_)]_2_ [Tp^Ph^* = hydro­bis­(3-phenyl­pyrazol-1-yl)(5-phenyl­pyrazol-1-yl)borate] (Cui *et al.*, 2010 ▸), where the aryl substituents on the bridging phosphinidenes cause steric congestion around the neodymium(III) centre; thus, the scorpionate undergoes a 1,2-boro­tropic shift. We believe that for borotropic shifting to occur there are a few conditions that need to be met: sufficient steric crowding around the metal centre, the conical angle of the scorpionate needs to be small and the metal centre needs to possess a certain Lewis acidity. The size of the conical angle is dependent on which scorpionate is used and must be relatively small for said scorpionate in order for shifting to occur. For the case of **1-Dy**, these conditions are met, whereas for **1-Ce**, the Lewis acidity is lower for the metal centre and the conical angles are slightly larger (Table 2 ▸). The Lewis acidity of lan­tha­nides is a direct consequence of ion size; thus, as the size of cerium(III) is larger [*cf.* dysprosium(III)], the Tp^2-Fu^ ligands sit further away from the metal centre and are not sterically congested.

**Figure 3:**
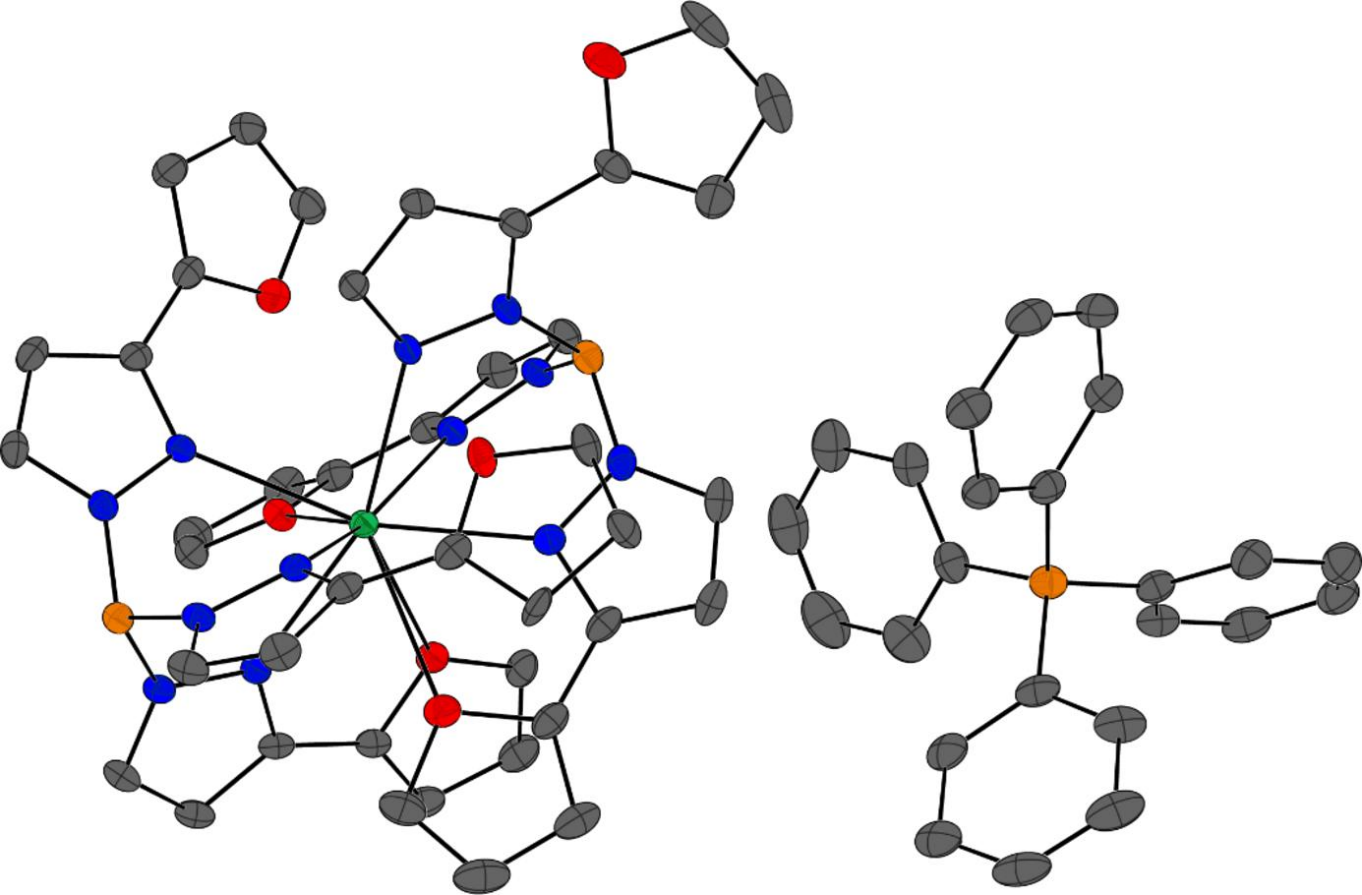
Solid-state structure of **2**, shown with 50% probability displacement ellipsoids. The colour codes are as in Fig. 1 ▸ and H atoms have been omitted for clarity. Only one conformation of the disordered furyl ring is shown, where the furyl torsion angle is 36.7 (6)°.

The solid-state structure of the cation [Dy(κ^4^-Tp^2-Fu^)(κ^5^-Tp^2-Fu^*)]^+^ in **2** is shown in Fig. 3 ▸, with the structural data presented in Table 2 ▸. One of the scorpionate ligands maintains its structure but resides much closer to the dysprosium(III) centre than in **1-Dy**, with a B⋯Dy distance of 3.601 (2) Å and a Dy—N_pz_ range of 2.375 (2)–2.558 (2) Å. This ligand also loses denticity adopting a κ^4^-binding mode, where two of the 2-furyl groups do not coordinate to the metal centre likely due to steric crowding, resulting in significantly larger furyl torsion angles of 23.5 (4) and 36.7 (6)°. The second furyl substituent in this ligand is disordered over two sites, where the additional furyl torsion angle is 149 (1)°, thus pointing away from the dysprosium centre (Fig. S4 in the supporting information). The one 2-furyl substituent in **2** that does coordinate has a shorter coordination bond of 2.542 (2) Å in com­parison with the 2-furyl substituents in **1-Dy**. The borotropic-shifted scorpionate Tp^2-Fu^* in **2** also resides much closer to the dysprosium(III) centre [Dy⋯B = 3.613 (4) Å and Dy—N_pz_ = 2.411 (2)–2.455 (2) Å] than in **1-Dy**, likely owing to the less sterically congested coordination environment. Due to the borotropic shift, one pyrazolyl substituent of the ligand is substituted in the 5-position, with the O_Fu_ atom pointing away from the metal centre; thus, the denticity of the ligand is reduced to κ^5^, as the remaining coordination bonds are maintained. The overall coordination number of **2** reduces to nine given the denticity of each ligand, with a capped square anti­prismatic (CSA) crystal field environment. The coordinating 2-furyl in Tp^2-Fu^* also present shorter Dy—O_Fu_ coordination bonds of 2.6400 (19) and 2.8418 (19) Å. Overall, the borotropic shifting of the Tp^2-Fu^ ligands produces a com­plex where the scorpionate ligands bind ‘tighter’ as the ligands reside closer to the dysprosium(III), which gives rise to a smaller nonlinear B⋯Dy⋯B angle of 155.94 (8)°.

## Conclusions

Through the synthesis of an early and mid-lan­tha­nide *bis*-Tp^2-Fu^ com­plex, we have demonstrated that 1,2-borotropic shifting of scorpionate ligands is likely dependent on the size, and hence Lewis acidity, of the lan­tha­nide ion and how close the 3-*R*-substituted scorpionate ligands reside, as seen in the literature. The hydrate synthetic route of **1-Ce** and air-stability of this com­plex aid this argument, while the anhydrous synthesis of **1-Dy** still results in the isolation of the more stable borotropic-shifted **2**. 

## Supplementary Material

Crystal structure: contains datablock(s) 1Ce, 1Dy, 2, global. DOI: 10.1107/S2053229624003115/ux3004sup1.cif

Structure factors: contains datablock(s) 1Ce. DOI: 10.1107/S2053229624003115/ux30041Cesup2.hkl

Structure factors: contains datablock(s) 1Dy. DOI: 10.1107/S2053229624003115/ux30041Dysup3.hkl

Structure factors: contains datablock(s) 2. DOI: 10.1107/S2053229624003115/ux30042sup4.hkl

Synthesis details and additional solid-state structural diagrams. DOI: 10.1107/S2053229624003115/ux3004sup5.pdf

CCDC references: 2330102, 2330103, 2330104

## Figures and Tables

**Table 1:** Experimental details Experiments were carried out at 100 K with Mo *K*α radiation using a Bruker D8 QUEST diffractometer. Absorption was corrected for by multi-scan methods (*SADABS*; Bruker, 2016 ▸). H atoms were treated by a mixture of independent and constrained refinement.

	**1-Ce**	**1-Dy**	**2**
Crystal data			
Chemical formula	[Ce(C_21_H_16_BN_6_O_3_)_2_](C_24_H_20_B)·2CH_2_Cl_2_	[Dy(C_21_H_16_BN_6_O_3_)_2_](C_24_H_20_B)·2CH_2_Cl_2_	[Dy(C_21_H_16_BN_6_O_3_)_2_](C_24_H_20_B)
*M*_r_	1451.59	1389.05	1304.12
Crystal system, space group	Monoclinic, *P*2_1_/*c*	Triclinic, *P* 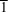	Triclinic, *P* 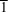
*a*, *b*, *c* (Å)	11.2742 (5), 27.1355 (13), 21.3644 (10)	12.1461 (7), 16.1883 (8), 17.1795 (10)	11.7200 (4), 11.9389 (5), 21.9139 (9)
α, β, γ (°)	90, 90.166 (2), 90	102.659 (2), 103.322 (2), 95.948 (2)	75.579 (2), 76.715 (1), 86.477 (1)
*V* (Å^3^)	6536.0 (5)	3164.6 (3)	2890.2 (2)
*Z*	4	2	2
μ (mm^−1^)	0.92	1.33	1.36
Crystal size (mm)	0.3 × 0.25 × 0.05	0.3 × 0.2 × 0.05	0.2 × 0.2 × 0.1

Data collection			
*T*_min_, *T*_max_	0.629, 0.746	0.610, 0.747	0.672, 0.747
No. of measured, independent and observed [*I* > 2σ(*I*)] reflections	596858, 16177, 14693	245023, 12926, 11705	230574, 11804, 10724
*R*_int_	0.056	0.058	0.052
(sin θ/λ)_max_ (Å^−1^)	0.667	0.625	0.625

Refinement			
*R*[*F* ^2^ > 2σ(*F* ^2^)], *wR*(*F* ^2^), *S*	0.032, 0.085, 1.06	0.044, 0.105, 1.08	0.028, 0.066, 1.18
No. of reflections	16177	12926	11804
No. of parameters	855	930	801
No. of restraints	0	14	0
Δρ_max_, Δρ_min_ (e Å^−3^)	1.64, −1.42	2.71, −1.74	2.69, −1.00

Computer programs: *APEX3* (Bruker, 2019 ▸), *SAINT* (Bruker, 2019 ▸), *SHELXT2014* (Sheldrick, 2015*a* ▸), *SHELXL2019* (Sheldrick, 2015*b* ▸) and *OLEX2* (Dolomanov *et al.*, 2009 ▸).

**Table 2:** Selected bond distances (Å) and angles (°), and Continuous Shape Measurement (CShM) values for coordination geometries calculated by *SHAPE2.1* (Pinsky *et al.*, 1998 ▸)

	**1-Ce**	**1-Dy**	**2**	
			**Tp^2-Fu^**	Tp^2-Fu^*
Ln⋯B	3.865 (3), 3.878 (3)	3.720 (7), 3.740 (6)	3.601 (2)	3.611 (3)
Ln—N_pz_	2.638 (2)–2.674 (2)	2.489 (4)–2.626 (3)	2.375 (2)–2.558 (2)	2.411 (2)–2.455 (2)
Ln—O_Fu_	2.809 (2)–2.890 (2)	2.755 (3)–3.181 (3)	2.5417 (17)	2.6400 (18), 2.8420 (18)
Furyl torsion angle	1.8 (3)–7.1 (3)	0.4 (6)–15.2 (6)	3.0 (3), 23.5 (4), 36.7 (6), 149 (1)*	3.0 (3), 6.7 (3), 150.8 (2)
Conical angle	42.45 (6)–43.27 (6)	41.7 (1)–44.6 (1)	41.04 (6)–45.26 (6)	41.74 (7)–42.78 (7)
B⋯Ln⋯B	179.30 (5)	173.1 (1)	155.94 (6)	
CShM (geometry)	0.358 (*I*_c_)	1.034 (*I*_c_)	1.422 (CSA)	

Notes: N_pz_ = coordinating pyrazolyl N atom; O^Fu^ = coordinating furyl O atom; furyl torsion angle = angle between pyrazolyl and furyl groups measured from N_pz_ to O_Fu_; *I*_c_ = icosa­hedral; CSA = capped square anti­prism. (*) Two conformations of the furyl group are present in **2** for one pyrazolyl substituent.
