# Supplementary material for: Borotropic shifting of the hydro­tris­[3-(2-furyl)pyrazol-1-yl]borate ligand in high-coordinate lan­tha­nide com­plexes
Source: Acta Crystallogr C Struct Chem. 2024 Apr 16;80(Pt 5):153–8. doi: 10.1107/S2053229624003115 (PMC11068059; doi:10.1107/S2053229624003115)
Supplement: Supplementary file 5 [file c-80-00153-sup5.pdf]

STRUCTURAL CHEMISTRY

Volume 80 (2024)

Supporting information for article:

Borotropic shifting of the [hydrotris(2′-furyl)pyrazol-1-yl]borate ligand in high-coordinate lanthanide complexes

Jarrod R. Thomas and Scott A. Sulway

S1. Experimental procedures

S1.1. General procedures

Starting material of $CeCl_3{\cdot}7H_2O$, $DyCl_3{\cdot}6H_2O$, $NaBPh_4$ and $NaBH_4$ were purchased commercially available sources. 3-(2'-furyl)-pyrazole was prepared via a literature procedure (Thomas *et al.*, 2021). Anhydrous $DyCl_3$ was synthesised by *in vacuo* thermal decomposition of ammonium chloride adduct. ^{1}H (400 MHz), ^{11}B (128 MHz), NMR Data was collected on a 400 MHz Brucker Advanced III at 298K fitted with BBFO temperature probes. ATR-Fourier transform Infrared (ATR-FTIR) spectra were collected on Thermo-Scientific Nicolet iS50 in ambient conditions. Elemental Analysis was carried out at the Mark Wainwright Analytical Centre (MWAC) XRF lab using a Elementar varioMACRO cube (CHN).

S1.1.1. Synthesis of sodium hydrotris(3-(2'-furyl)-pyrazolyl-1-yl)borate, NaTp$^{2\text{-}Fu}$

Synthesis of scorpionate ligands follow general literature procedures. Freshly sublimed 3-(2'-furyl)-pyrazole (5.68 g, 42.3 mmol) and $NaBH_4$ (0.500 g, 13.2 mmol) were thoroughly mixed before being placed under an inert atmosphere. The solid mixture was heated to 200°C, to which the pyrazole reagent became molten, and evolution of hydrogen gas was observed. This temperature was maintained for roughly 20 hours before being cooled and washed with copious amounts of toluene. During washing, the mixture was sonicated to aid breaking up the solid and dissolve unreacted pyrazole reagent. Product was filtered and washed with toluene and warm hexane yielding a white powder (4.61 g, 10.62 mmol, 80%). Elemental analysis calculated for $C_{21}H_{16}N_6O_3B_1Na_1$: C 58.09, H 3.71, N 19.36; found: C 57.92, H 3.39, N 19.04. FTIR (ATR, powder): $\bar{\nu}$ = 2422 (s, ν_{BH}) cm^{-1}. ^{1}H NMR (400 MHz, d_6-acetone, 298 K): δ = 7.68 (d, 3H, $^{3}J_{HH}$ = 2.16 Hz, pzH), 7.44 (dd, 3H, $^{3}J_{HH}$ = 1.80 Hz, $^{4}J_{HH}$ = 0.72 Hz, FuH), 6.59 (dd, 3H, $^{3}J_{HH}$ = 3.30, $^{4}J_{HH}$ = 0.70 Hz, FuH), 6.42 (dd, 3H, $^{3}J_{HH}$ = 3.30 Hz and 1.82 Hz, FuH), 6.33 (d, 3H, $^{3}J_{HH}$ = 2.20 Hz, pzH) ppm; ^{11}B NMR (128 MHz); δ = -1.84 (d, $^{1}J_{BH}$ = 101.8 Hz, BH borate) ppm.

S1.1.2. Synthesis of [Ce(Tp$^{2\text{-}Fu}$)$_2$](BPh$_4$)·(CH$_2$Cl$_2$)$_3$ (1-Ce)·(CH$_2$Cl$_2$)$_3$)

$CeCl_3{\cdot}7H_2O$ (0.19 g, 0.51 mmol) and NaTp$^{2\text{-}Fu}$ (0.52 g, 1.2 mmol) were stirred in MeOH (20 ml) for 30 minutes, before a $NaBPh_4$ (0.18 g, 0.53 mmol) in MeOH (3 ml) was added. After stirring for 2 hours the precipitate produced was collected by filtration and washed with MeOH (2 x 5 ml), yielding a white powder of **1-Ce** (0.53 g, 0.41 mmol, 80%). Single crystals suitable for X-ray diffraction of **1-Ce**·(CH_2Cl_2) were grown from a solution of **1-Ce** in CH_2Cl_2 layered with pentane. Elemental analysis indicates likely loss of lattice solvent after isolation (approximately 2.5 CH_2Cl_2); elemental analysis calculated for (**1-Ce**) $C_{66}H_{52}B_3Ce_1N_{12}O_6$: C 61.85, H 4.09, N 13.11; (**1-Ce**)·$(CH_2Cl_2)_{0.5}$: C 60.27, H 4.11, N 12.68; found: C 60.81, H 4.33, N 12.98. FTIR (ATR, microcrystalline): 3130 (w), 3118 (w), 3050 (w), 3036 (w), 3020 (w), 3010 (w), 2992 (w), 2501 (w), 2481 (w), 1578 (w), 1524 (s), 1492 (s), 1478 (w), 1456 (s), 1424 (w), 1400 (w), 1388 (s), 1351 (s), 1342 (s), 1259 (w), 1218 (s), 1199 (s), 1156

(s), 1113 (s), 1061 (s), 1049 (s), 1032 (w), 1014 (s), 1001 (s), 969 (s), 892 (s), 880 (s), 834 (w), 811 (s), 790 (s), 778 (s), 765 (s), 729 (s), 704 (s), 656 (s), 633 (s), 623 (w), 611 (s) cm^{-1}.

S1.1.3. Synthesis of $[Dy(Tp^{2\text{-}Fu})_2](BPh_4)\cdot CH_2Cl_2$ (1-Dy·CH_2Cl_2) and $[Dy(\kappa^4\text{-}Tp^{2\text{-}Fu})(\kappa^5\text{-}Tp^{2\text{-}Fu*})](BPh_4)$ (2)

$DyCl_3$ (0.269 g, 1.00 mmol), $NaTp^{2\text{-}Fu}$ (0.868 g, 2.00 mmol) and $NaBPh_4$ (0.324 g, 1.00 mmol) where charged in a flask and placed under an inert atmosphere. To the powders dry and degassed THF (15 ml) was added, and the solution was allowed to stir for 3 days producing a yellow solution was a white precipitate. The solvent was removed *in vacuo* and replaced with CH_2Cl_2 (10 ml) before having the solids removed by cannular filtration and the filtrate layered with pentane (7 ml). After 1-day, single crystals of **1-Dy**·CH_2Cl_2 had grown which was collected on a glass frit and washed with pentane (2 x 10 ml) (0.131 g, 0.0935 mmol, 9%). Elemental analysis calculated for $C_{74}H_{60}B_3Dy_1N_{14}O_7Cl_2$: C 58.35, H 3.97, N 12.87; found: C 57.29, H 3.25, N 11.06. FTIR (ATR, microcrystalline), we attribute the variation being due to the pyrazole fragmentation as indicated by the X-ray diffraction data still being present in the crude material: 3127 (br, w), 3055 (w), 3036 (w), 3000 (w), 2983 (w), 2488 (br, w), 1580 (w), 1519 (s), 1494 (s), 1479 (w), 1454 (s), 1426 (w), 1390 (s), 1351 (s), 1341 (s), 1262 (w), 1222 (s), 1201 (s), 1161 (s), 1115 (s), 1049 (s), 1014 (s), 1000 (s), 972 (s), 892 (s), 845 (w), 809 (s), 788 (s), 776 (s), 730 (s), 702 (s), 657 (s), 633 (w), 624 (w), 611 (s) cm^{-1}. The mother liquor and washes were kept which produced crystals of **2** overnight (0.221 g, 0.169 mmol, 6%). Elemental analysis calculated for $C_{66}H_{52}B_3Dy_1N_{12}O_6$: C 60.78, H 4.02, N 12.89; found: C 60.52 H 3.98, N 12.77. FTIR (ATR, microcrystalline): 3131 (w), 3118 (w), 3053 (w), 3037 (w), 3020 (w), 2992 (w), 2983 (w), 2963 (w), 2487(br, w), 1579 (w), 1522 (s), 1495 (s), 1478 (w), 1456 (s), 1424 (w), 1492 (w), 1352 (s), 1341 (s), 1260 (w), 1223 (s), 1204 (s), 1162 (s), 1114 (s), 1064 (s), 1050 (s), 1014 (s), 1001 (s), 973 (s), 893 (s), 882 (s), 847 (w), 808 (s), 792 (s), 779 (s), 767 (s), 729 (s), 703 (s), 658 (s), 633 (s), 624 (w), 612 (s) cm^{-1}.

S2. X-Ray Crystallography Images

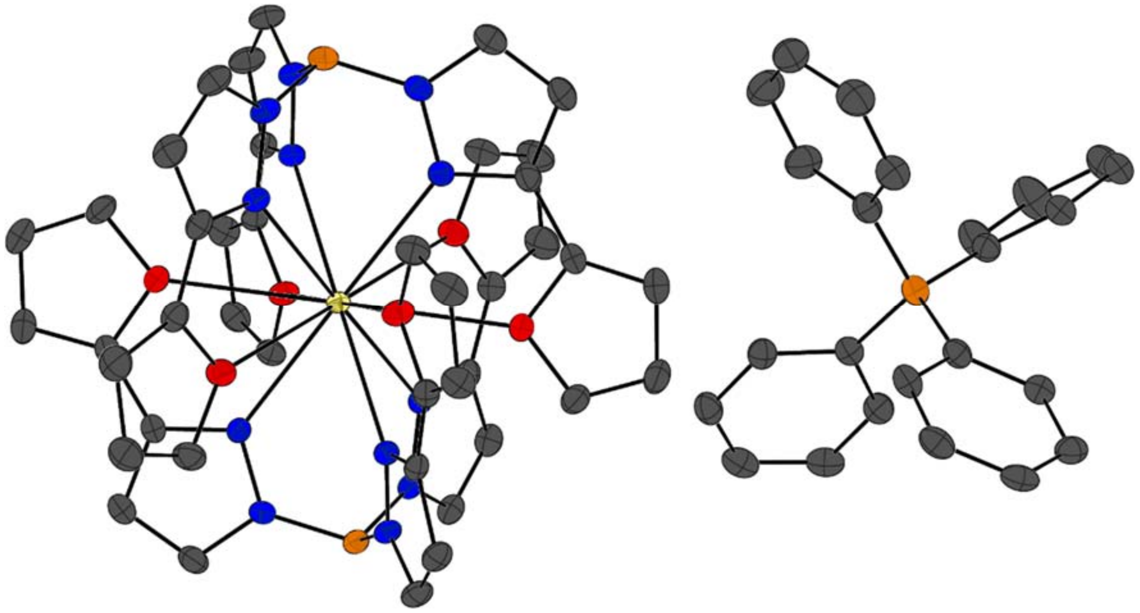

Figure S1 Solid-state structure of **1-Ce**, shown with 50% ellipsoids. Cerium(III) = cream, oxygen = red, nitrogen = blue, carbon = grey, boron = orange, hydrogen atoms and lattice solvents have been omitted for clarity.

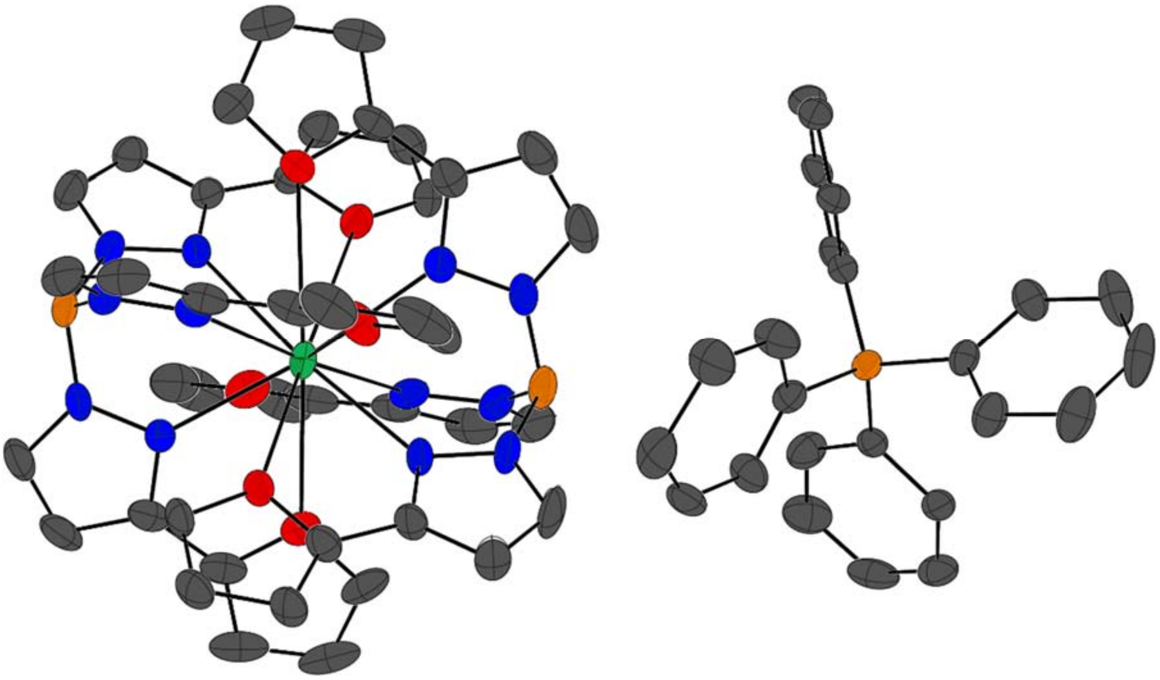

Figure S2 Solid-state structure of **1-Dy**, shown with 50% ellipsoids. Dysprosium(III) = cream, oxygen = red, nitrogen = blue, carbon = grey, boron = orange, hydrogen atoms and lattice solvents have been omitted for clarity. Only one confirmation of the BPh_4 anion is shown for **1-Dy**.

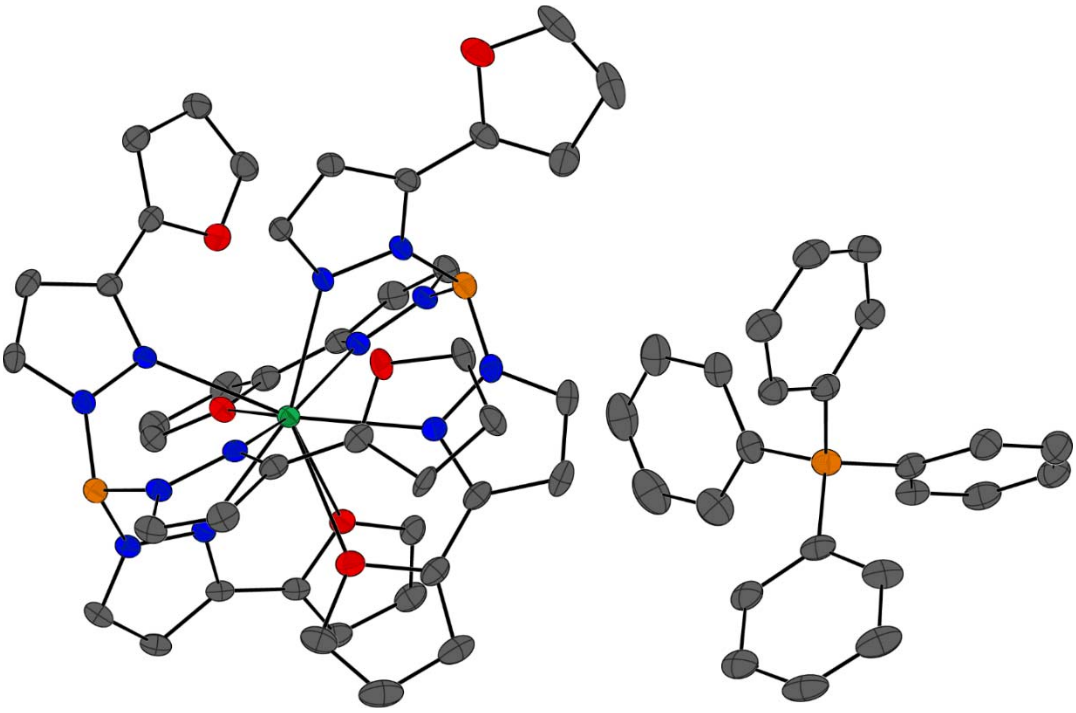

Figure S3 Solid-state structure of **2**, shown with 50% ellipsoids. Dysprosium(III) = cream, oxygen = red, nitrogen = blue, carbon = grey, boron = orange, hydrogen atoms have been omitted for clarity. Only one conformation of the disordered furyl ring is shown, where the furyl torsion angle = 36.7(6)°.

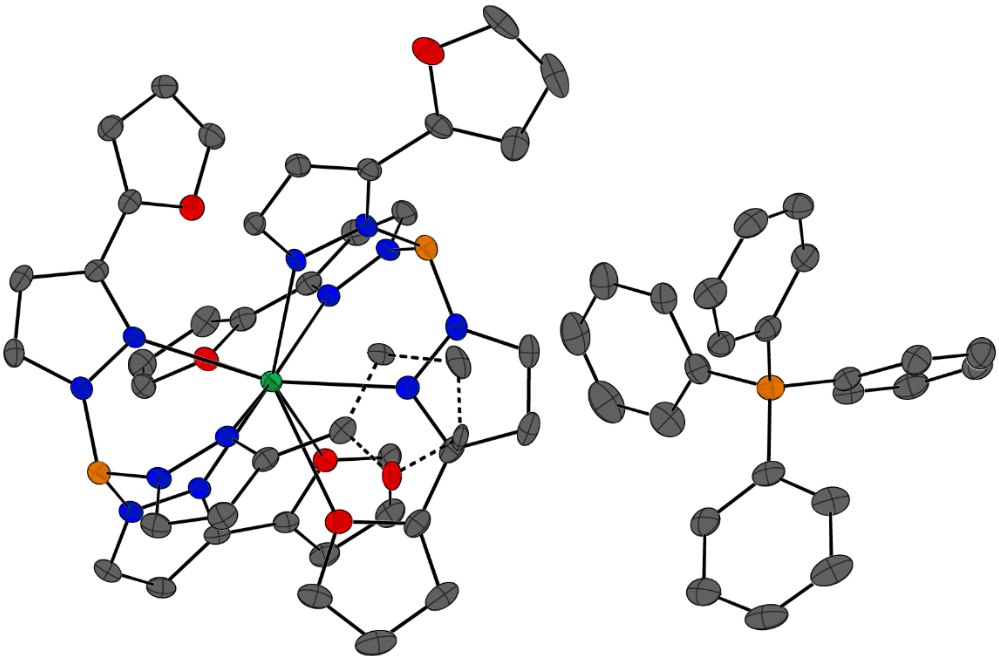

Figure S4 Solid-state structure of **2**, shown with 50% ellipsoids. Dysprosium(III) = cream, oxygen = red, nitrogen = blue, carbon = grey, boron = orange, hydrogen atoms have been omitted for clarity. Only one conformation of the disordered furyl ring is shown, where the furyl torsion angle = 149(1)°.
